# Efficacy of acupuncture for stroke-associated pneumonia: a systematic review and meta-analysis

**DOI:** 10.3389/fmed.2025.1440121

**Published:** 2025-03-03

**Authors:** Kaihan Su, Xiaoyu Wang, ShiYin Zhang, Jiantong Wu, Yuqi Chen, Lianjun Yin, Haunan Li, Jingui Wang

**Affiliations:** ^1^Department of Tuina, First Teaching Hospital of Tianjin University of Traditional Chinese Medicine, Tianjin, China; ^2^National Clinical Research Center for Chinese Medicine Acupuncture and Moxibustion, Tianjin, China; ^3^Department of Acupuncture and Tuina Rehabilitation, Kunshan Affiliated Hospital of Nanjing University of Traditional Chinese Medicine, Kunshan, China; ^4^Department of Rehabilitation Medicine, The Third Affiliated Hospital of Southern Medical University, Guangzhou, China

**Keywords:** acupuncture, stroke-associated pneumonia, meta-analysis, systematic review, SAP

## Abstract

**Objectives:**

This study aims to systematically evaluate the efficacy of acupuncture on stroke-associated pneumonia (SAP).

**Methods:**

English and Chinese databases were searched from their inception until 15 March 2024 to collect randomized controlled trials (RCTs). The risk of bias was assessed using Cochrane collaboration tools. RevMan 5.4.0 software was used to analyze the included studies, and the Grades of Recommendations, Assessment, Development, and Evaluation (GRADE) assessment was used to evaluate the quality of the study outcomes.

**Results:**

16 studies involving 1,125 patients were included in this meta-analysis. Compared with the control group, the results showed that acupuncture significantly improved the effective rate [RR = 1.20, 95% CI (1.13, 1.27), *P* < 0.00001] and reduced the level of white blood cells (WBC) [MD = −6.52, 95% CI (−8.31, −4.73), *P* < 0.00001], C reactive protein (CRP) [MD = −6.50, 95% CI (−9.97, −3.03), *P* = 0.0002], neutrophil percentage (Neu%) [MD = −6.66, 95% CI (−8.96, −4.36), *P* < 0.00001], and procalcitonin (PCT) [MD = −0.81, 95% CI (−1.21, −0.40), *P* < 0.0001]. Additionally, acupuncture therapy shortened the duration of coughing [MD = −3.22, 95% CI (−4.73, −1.72), *P* < 0.0001], duration until disappearance of rales [MD = −3.99, 95% CI (−6.44, −1.54), *P* = 0.001], and duration of antibiotic use [MD = −4.51, 95% CI (−5.46, −3.57), *P* < 0.00001]. It also reduced the clinical pulmonary infection score (CPIS) [MD = −1.71, 95% CI (−2.71, −0.71), *P* = 0.0008] and National Institute of Health Stroke Scale (NIHSS) [MD = −3.93, 95% CI (−5.78, −2.09), *P* < 0.00001]. Moreover, acupuncture therapy increased the forced vital capacity (FVC) [MD = 0.46, 95% CI (0.02, 0.89), *P* = 0.04] and Forced Expiratory Volume in One Second (FEV_1_) [MD = 0.49, 95% CI (0.14, 0.84), *P* = 0.006].

**Conclusion:**

This study found that acupuncture has a positive effect in treating SAP. However, owing to the low-quality evidence, more rigorous studies are needed in the coming years to confirm these findings.

**Systematic review registration:**

https://www.crd.york.ac.uk/prospero/display_record.php?ID=CRD42023462846, identifier CRD42023462846.

## 1 Introduction

According to the Global Burden of Disease Study 2021 (1), stroke is the third leading cause of death worldwide (1). As revealed by the Global Burden of Disease Study 2016, stroke not only causes death but also disability, profoundly affecting the health and quality of life of millions of people (2). Infection is both a risk factor for stroke and a determinant of post-stroke outcomes (3). The patient’s immune response is suppressed following a stroke, adversely influencing survival and recovery (4). Pneumonia is the most common type of infection and has a greater impact on clinical outcomes (5). Additionally, approximately 42% of stroke patients experience post-stroke dysphagia (PSD), which heightens the risk of silent aspiration and penetration, potentially resulting in aspiration pneumonia (6). Stroke-associated pneumonia (SAP) is a frequent complication that arises from lower respiratory tract infections within the first week following a stroke (7), affecting approximately 14.3% of stroke patients (8). Moreover, it is closely associated with a variety of complications such as gastrointestinal bleeding (8.35%), bedsores (5.31%), deep vein thrombosis (4.27%), epileptic seizures (3.96%), urinary tract infections (3.34%), atrial fibrillation/flutter (3.17%), and recurrent stroke (2.65%) (9). It elevates mortality rates for up to one year, extends the duration of hospital stays, and deteriorates functional outcomes at discharge (10, 11).

SAP plays a vital role in the development of various complications after stroke, emphasizing the need for integrative therapy to help patients get better outcomes. Antibiotics remain the primary medication for treating pneumonia (12). However, their use failed to improve the prognosis for stroke patients (13). In addition, the current evidence does not support using antibiotics or immunomodulatory approaches as a preventative measure to prevent pneumonia in stroke patients with dysphagia, even if they are at a high risk of aspiration (13, 14). Even though Angiotensin Converting Enzyme (ACE) inhibitors were associated with lower pneumonia-related mortality, the data is not convincing (15). Similarly, while metoclopramide could reduce the incidence of pneumonia after stroke, it did not decrease mortality rates and lack of robust evidence (16). Consequently, given the limitations of conventional treatment (CT) in improving the prognosis of SAP, exploring effective alternative therapies for SAP is essential. Research has proven that dysphagia screening and rehabilitation, feeding modification, oral care, airway management, position management, and Traditional Chinese Medicine (TCM) nursing techniques have significant effects on the prevention of SAP (17).

Acupuncture has been widely used in the recovery after stroke and has shown favorable results by regulating various mechanisms within the central nervous system (CNS) (18). Moreover, acupuncture therapy has been shown to reduce the inflammatory response by affecting the regulation of multiple cytokines, making it an effective potential treatment for respiratory disease (19). In recent years, several small-sample randomized controlled trials (RCTs) on acupuncture for SAP have been conducted domestically and internationally (20–35). While these studies have confirmed the positive effects of acupuncture on SAP, due to the varying outcome measures among these studies, there is still lacking robust and high-quality evidence to support these findings.

According to our knowledge, no high-quality systematic review or meta-analysis currently provides a comprehensive summary of this topic. Therefore, further research is needed to fully understand this subject. This meta-analysis significantly enhances our understanding of the role of acupuncture in SAP. We hope that the findings of this study will capture the interest of healthcare professionals, researchers, and patients alike.

## 2 Materials and methods

This systematic review and meta-analysis was conducted according to the Preferred Reporting Items for Systematic Review and Meta-Analysis (PRISMA) guidelines (36). The study protocol was registered in PROSPERO (number CRD42023462846).

### 2.1 Search strategy

We searched PubMed, Embase, Sinomed, the Cochrane Library, Web of Science, China National Knowledge Infrastructure (CNKI), Chinese Scientific Journal Database (VIP), and Wanfang Data database. The search covered the period from the inception of these databases to 15 March, 2024. The keywords and search terms were imposed: “stroke,” “pneumonia,” “stroke associated pneumonia,” “acupuncture,” and “RCT.” More search strategies are available in Supplementary File 1.

### 2.2 Inclusion and exclusion criteria

Inclusion criteria:

1. Types of participants: Individuals meeting the diagnostic criteria for SAP were diagnosed by the “Chinese expert consensus on the diagnosis and treatment of stroke-associated pneumonia” (37) or “Diagnosis of stroke-associated pneumonia: recommendations from pneumonia in stroke consensus group” (7), with no restrictions on nationality, ethnicity, age, gender, or other demographic factors.
2. Types of intervention: The treatment group receives acupuncture and CT.
3. Types of comparison: The control group receives CT without acupuncture therapy.

4. Types of outcomes: The primary outcome measure was the effective rate. The secondary outcomes were the inflammatory markers [C-reactive protein (CRP), procalcitonin (PCT), white blood cell count (WBC), neutrophil percentage (Neu%)], pulmonary symptoms forced vital capacity (FVC), forced expiratory volume in 1 s (FEV_1_), cough duration, duration until disappearance of rales, and clinical pulmonary infection score (CPIS), national institute of health stroke scale (NIHSS), duration of antibiotic use and incidence of adverse reactions.

5. Types of study: RCTs.

Exclusion Criteria:

1. Reviews, basic experiments, and reports.
2. Unpublished or duplicate publications.
3. Studies with missing data.
4. The control group receives acupuncture therapy.

### 2.3 Study selection and data extraction

Two reviewers (KHS and XYW) independently reviewed the titles and abstracts of identified studies to exclude unrelated studies that did not fit the subject of the study, and disagreements were resolved through discussion with the third reviewer (JTW). Subsequently, a researcher (KHS) extracted the information required for this study from the selected articles into Excel 2016, including the first author, publication year, average age, randomization method, sample size, intervention method, adverse reactions, allocation concealment, outcome measures, and their data integrity information. After data extraction, all data were checked by another investigator (XYW), and discrepancies were discussed and resolved by the third reviewer (JTW).

### 2.4 Statistical analysis

Review Manager 5.4.0 and Stata 14.0 were used for systematic evaluation and meta-analysis in this study. Continuous data was represented by mean difference (MD) with a corresponding 95% confidence interval (CI), while binary data was represented by risk ratio (RR) with a corresponding 95% confidence interval. Heterogeneity between studies was assessed using the chi-square test and I-squared (*I*^2^) index. A fixed effects model was employed if the *P* ≥ 0.05 and *I*^2^ ≤ 50%; otherwise, a random effects model was chosen. Statistical significance was denoted by a significance level of *P* ≤ 0.05. If the number of studies was > 10 for comparison, we assessed the possibility of publication bias using funnel plots and assessed funnel plot asymmetry using Egger’s regression test (*P* < 0.1).

### 2.5 Risk of bias assessment

The Cochrane bias risk assessment tool (38) was used by two researchers (KHS and XYW) to evaluate the risk of bias in the included literature, and disagreements were resolved through discussion with the third reviewer (JTW). This evaluation covered aspects such as randomisation processes, allocation concealment, blinding techniques, completeness of data, reporting selectivity, and other bias risks. The outcomes of this evaluation were classified into three levels: “high risk,” “unclear,” or “low risk.”

### 2.6 GRADE evidence quality assessment

The Grades of Recommendations, Assessment, Development, and Evaluation (GRADE) assessment (39) was used to evaluate the quality of the study outcomes. The quality of the evidence was categorized as “high,” “medium,” “low,” or “very low” depending on the study’s findings Grade. Factors affecting the grade of evidence include risk of bias, inconsistency, indirectness, precision, and publication bias. Details of the GRADE evidence quality assessment are presented in Table 1.

**TABLE 1:** GRADE of evidence of outcomes of the included trials.

Outcomes	Certainty assessment						No. of patients		Effect sizes	Certainty
	**No. of trials**	**Risk of bias**	**Inconsistency**	**Indirectness**	**Imprecision**	**Publication bias**	**Intervention**	**Control**	**RR or SMD (95% CI)**	
Effective rate	7	Serious	No serious	No serious	No serious	Undetected	418	418	RR1.47 (1.03, 2.09)	⊕⊕⊕⊖ Moderate
CRP	7	Serious	Serious	No serious	No serious	Undetected	288	288	MD −6.50 (−9.97, −3.03)	⊕⊕⊖⊖ Low
PCT	5	Serious	Serious	No serious	Serious	Undetected	208	208	MD −0.81 (−1.21, −0.40)	⊕⊖⊖⊖ Very low
WBC	4	Serious	Serious	No serious	No serious	Undetected	363	363	MD −6.52 (−8.31, 4.73)	⊕⊖⊖⊖ Low
Neu%	3	Serious	Serious	No serious	Serious	Undetected	140	140	RR −6.66 (−8.96, −4.36)	⊕⊖⊖⊖ Very low
Cough duration	5	Serious	Serious	No serious	Serious	Undetected	182	182	MD −3.22 (−4.73, −1.72)	⊕⊖⊖⊖ Very low
Duration until disappearance of rales	4	Serious	Serious	No serious	Serious	Undetected	147	147	MD −3.99 (−6.44, −1.54)	⊕⊖⊖⊖ Very low
CPIS	5	Serious	Serious	No serious	Serious	Undetected	162	162	MD −1.71 (−2.71, −0.71)	⊕⊖⊖⊖ Very low
FVC	3	Serious	Serious	No serious	Serious	Undetected	110	110	MD 0.46 (0.02, 0.89)	⊕⊖⊖⊖ Very low
FEV1	2	Serious	Serious	No serious	Serious	Undetected	80	80	MD 0.49 (0.14, 0.84)	⊕⊖⊖⊖ Very low
NIHSS	4	Serious	Serious	No serious	Serious	Undetected	178	178	MD −3.93 (5.78, −2.09)	⊕⊖⊖⊖ Very low
Duration of antibiotic use	2	Serious	No serious	No serious	Serious	Undetected	67	67	MD −4.51 (−5.46, −3.57)	⊕⊕⊖⊖ Low

RR, risk ratios; MD, mean difference; CI, confidence interval; Risk of bias: serious, study with unclear risk of bias; Inconsistency: Serious, *I*^2^ > 50%. Indirectness: no indirectness of evidence was found in any study. Imprecision (based on sample size): Serious, *n* < 500 participants. Publication bias: Undetected due to the number of trials less than the recommended arbitrary minimum number of 10. GRADE Working Group grades of evidence. High certainty: We are confident that the true effect is close to the effect estimates. Moderate certainty: We are moderately confident in the effect estimate. The true effect is likely close to the effect’s estimate, but it may be substantially different. Low certainty: Our confidence in the effect estimate is limited. The true effect may be significantly different from the estimate of the effect. Very low certainty: We have very little confidence in the effect estimate. The true effect is likely to be substantially different from the estimate of the effect.

## 3 Results

### 3.1 Study selection

**FIGURE 1:**
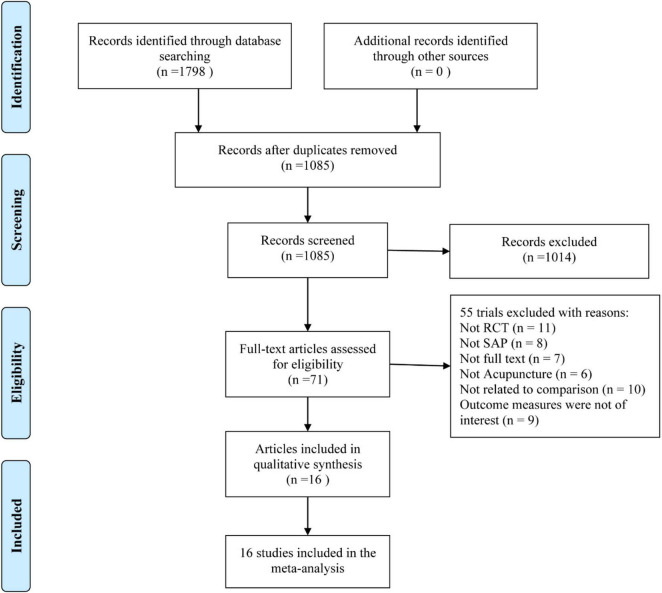
PRISMA flow chart.

Initially, a total of 1,798 studies were obtained from the database. Subsequently, 1,085 duplicate records were removed, and 1,014 irrelevant records were excluded based on titles and abstracts. After screening the complete text, 55 trials were further excluded. Ultimately, 16 trials (20–35) were included. Details of the selection process are presented in Figure 1.

### 3.2 Study characteristics

**FIGURE 2:**
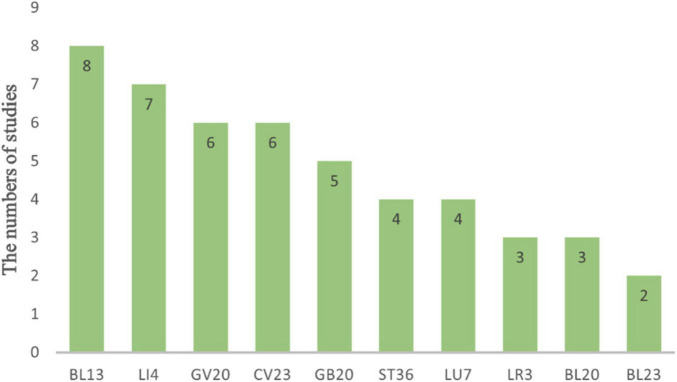
Acupoints selection of included RCTs.

The studies were conducted by Chinese researchers and published from 2014 to 2023 in China. A total of 16 trials (20–35) involving 1,125 patients were included in the study. There were 613 patients in the experimental group and 612 patients in the control group. In the control group, CT was used alone in 8 trials (23, 26, 29, 31–35), combined with swallowing rehabilitation in 2 trials (25, 30), with extracorporeal diaphragm pacing in 1 trial (27), with breathing training in 4 trials (21, 22, 27, 28), and with both breathing training and ultrashort wave electrotherapy in 1 trial (24). The detailed intervention methods for the control group are provided in Supplementary File 2. In the experimental groups, patients were treated with manual acupuncture (MA) in 13 trials (20–24, 27–32, 34, 35); Electroacupuncture (EA) in 2 trials (25, 26); and acupuncture point embedding (APE) in one trial (33). The most frequent retention time of MA and EA was 30 min (21, 23–32, 34), and 1 MA was 15 min (20), 1 MA was 20 min (22), and 1 MA only aimed to achieve “De qi” without retaining the needle (35). Additionally, 1 trial required the APE to be done once a week without mention of retention time (33). The treatment period varied from 10 to 28 days, and the most frequent treatment period was 14 days (20–22, 26, 28–35). All trials reported acupoints, and the top 5 acupoints with the highest frequency were BL13 (eight times) (21, 22, 25, 26, 29, 30, 33, 34), LI4 (seven times) (20, 21, 24, 27, 28, 31, 34), GV20 (six times) (20, 24, 25, 28, 31, 34), CV23 (six times) (20, 23, 24, 28, 34, 35), and GB20 (five times) (20, 24, 25, 28, 29), as shown in Figure 2. Adverse events were not mentioned. Detailed information regarding study design, participant demographics, and measured outcomes can be found in Table 2.

**TABLE 2:** Characteristics of included RCTs.

References	Sample size (E/C)	Age (E/C)	Gender (M/F)	Stroke type (I/H)	Course (E/C) d	Experimental group	Acupuncture points	Needling time	Control group	Duration	Outcome
Zhang et al. (20)	50/50	E:54.38±7.06 C:55.22±7.32	E:24/26 C:25/25	NR	NR	MA plus C	EX-HN5, GB20, LR3, EX-HN1, LI4, CV23, GV20, EX-HN3	1 session/day, 15 min/session	CT plus breathing training	14 days	Effective rate, CRP, PCT, WBC, cough duration, FVC, FEV1
Lin et al. (21)	30/30	E:63.9±1.1 C:63.8±0.8	E:20/10 C:19/11	NR	E:2.6±0.5 C:2.6±0.4	MA plus C	BL13, LI4, LU7	6 sessions/week, 30 min/session	CT plus breathing training	14 days	Effective rate, CRP, WBC, Neu%, PCT, CPIS, NIHSS
Guo et al. (22)	60/60	E:63.58±13.55 C:63.26±13.41	E:35/25 C:33/27	NR	NR	MA plus C	GV14, BL12, BL13	1 session/day, 20 min/session	CT plus breathing training	14 days	Effective rate
Guo et al.(23)	37/37	E:65.61±11.75 C:66.08±10.63	E:21/16 C:18/19	NR	NR	MA plus C	CV23	1 session/day, 30 min/session	CT	10 days	Duration of antibiotic use, CPIS, cough duration, duration until disappearance of rales
Ma et al. (24)	48/48	E:68.03±7.18 C:67.87±6.94	E:37/11 C:32/16	E:28/20 C:31/17	E:10.51±2.11 C:10.33±2.02	MA plus C	GB20, LR3, LI4, GV20, CV23	1 session/2 day, 30 min/session	CT plus breathing training plus ultrashort wave electrotherapy	28 days	Effective rate, CRP, WBC, PCT
Yuan et al.(25)	40/40	E:70±7 C:71±5	E:25/15 C:24/16	E:37/3 C:38/2	E:3.7±1.3 C:3.8±1.4	EA plus C	BL13, GV14, BL12, GB20, GV20	1 session/day, 30 min/session	CT plus Swallowing rehabilitation	10 days	Effective rate, CRP, WBC, Neu%, cough duration, duration until disappearance of rales, NIHSS
Tang and Cui (26)	30/30	E:61±17.25 C:57±19.61	E:16/14 C:14/16	NR	E:4.9±2.65 C:5.62±7.31	EA plus C	BL13, BL23, BL20, LU1, LU9, LU7	1 session/day, 30 min/session	CT	14 days	Effective rate, PCT, WBC, CPIS
Zhang et al. (27)	30/30	E:60.37 C:59.06	E:21/9 C:23/7	NR	NR	MA plus C	ST36, GB35, LI4, LI11	6 sessions/week, 30 min/session	CT plus Extracorporeal diaphragm pacing	15 days	Effective rate, PCT, WBC, FVC, FEV1
Fan (28)	20/20	E:67.43±3.62 C:67.24±3.51	E:13/7 C:12/8	NR	E:20.48±3.12 C:20.16±3.01	MA plus C	GB20, LR3, LI4, GV20, CV23	1 session/day, 30 min/session	CT plus breathing training	14 days	CRP, PCT, WBC, cough duration, the disappearance time of rales
Xia et al. (29)	73/73	E:64±3.2 C:62.8±2.7	E:49/24 C:47/26	E:58/15 C:61/12	E:8.6±2.1 C:9.0±2.0	MA plus C	BL13, LU1, LU9, GB20, LU7, ST40, ST36	1 session/day, 30 min/session	CT	14 days	NIHSS
Wu et al. (30)	30/30	E:59.90±11.34 C:61.34±10.73	E:22/8 C:21/9	E:21/9 C:18/12	NR	MA plus C	BL13, BL23, BL20	1 session/day, 30 min/session	CT plus Swallowing rehabilitation	14 days	Effective rate, CRP, WBC, CPIS, FVC
Luo et al. (31)	35/35	E:61±11 C:63±11	E:20/15 C:18/17	NR	E:9.05±1.88 C:9.03±1.87	MA plus C	GV20, LI15, LI11, ST36, TE5, LI4, GB31, GB34, BL35, BL60	6 sessions/week, 30 min/session	CT	14 days	NIHSS, CPIS
Liu et al. (32)	30/29	E:53.6±4.4 C:63.15±10.6	E:23/7 C:21/8	E:22/8 C:23/6	E:12.9±5.9 C:10.8±6	MA plus C	RN23, KI5, GB3, KI1	1 session/day, 30 min/session	CT	14 days	Effective rate
Gu et al. (33)	35/35	E:71±5 C:69±6	E:20/15 C:18/17	E:24/11 C:21/14	E:3.1±1.7 C:3.2±1.9	APE plus C	BL13, BL20	1 session/week	CT	14 days	Effective rate, CRP, WBC, Neu%
Liang (34)	35/35	E:63.15±10.6 C:62.36±10.77	E:21/14 C:19/16	E:20/15 C:21/14	E:4.32±1.71 C:4.29±1.56	MA plus C	BL13, LI4, LU7, CV12, CV22, ST36, GV20, CV23	6 sessions/week, 30 min/session	CT	14 days	Effective rate, CRP, WBC, Neu%, cough duration, duration until disappearance of rales
Gao and Su(35)	30/30	E:51±4 C:53±2	E:20/10 C:23/7	E:26/4 C:27/3	E:15.4±5.2 C:17± 6	MA plus C	ST9, CV23, SP6	6 sessions/week, De qi	CT	14 days	Effective rate, duration of antibiotic use

E, experiment group; C, control group; M, male; F, female; NR, not reported; MA, manual acupuncture; APE, acupuncture point embedding; CT, conventional therapy; H, hemorrhagic stroke, I, ischemic stroke.

### 3.3 Risk-of-bias

**FIGURE 3:**
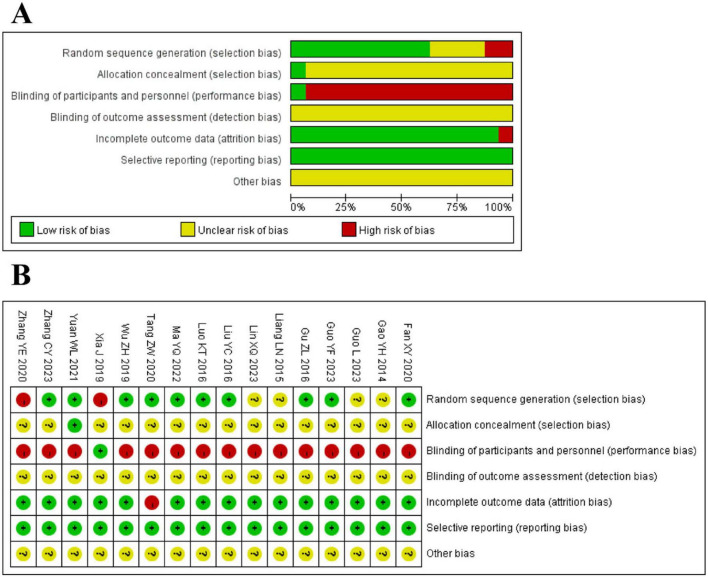
**(A)** The graph of risk of bias. **(B)** The summary of the risk of bias.

We assessed the risk of bias for all 16 trials (20–35), and the detailed assessment data are presented in Figure 3. For randomized sequence generation, 10 trials (20, 22, 24–26, 28, 30, 31, 33) were at low risk of bias, 2 trials (27, 29) were at high risk, and 4 trials (21, 23, 34, 35) were at unclear risk of bias. For allocation concealment, 15 trials (20–24, 26–35) were at unclear risk of bias in this area, and 1 trial (25) was at low risk of bias. 1 trial (29) reported blinding of participants and researchers, whereas other trials (20–28, 30–35) did mention this aspect, thereby leaving unclear risk of bias. The risk of bias in the blinding of outcome assessments was unclear in all studies. Regarding incomplete outcome data, 15 trials (20–25, 27–35) had a low risk of bias, and 1 trial (26) had a high risk of bias. In terms of selective reporting, all 16 trials (20–35) were at low risk of bias. For other biases, the risk of bias for all 16 trials (20–35) was unclear.

### 3.4 Meta-analysis

#### 3.4.1 Effective rate

**FIGURE 4:**
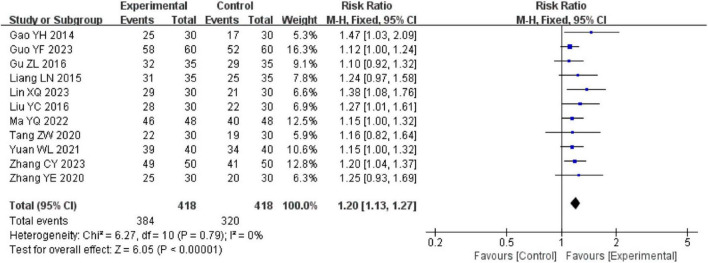
The forest of the antibiotic use duration compared to acupuncture plus CT vs. CT.

11 trials using a fixed effect model reported the effective rate between the intervention and control groups. The results demonstrated that the intervention group showed superior effectiveness than the control group [RR = 1.20, 95% Cl (1.13, 1.27), *P* < 0.00001], with no significant heterogeneity detected (*I*^2^ = 0%, *P* = 0.79) (Figure 4).

#### 3.4.2 Inflammatory markers in blood routine

##### C-reactive protein (CRP) levels

**FIGURE 5:**
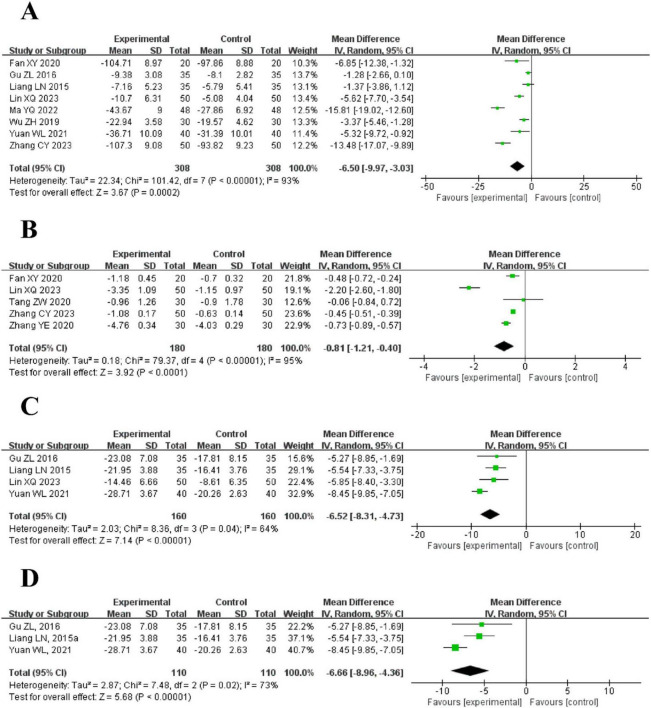
The forest plot of inflammatory markers in blood routine compared to acupuncture plus CT vs. CT. **(A)** C-Reactive Protein (CRP) Levels. **(B)** Procalcitonin (PCT) Levels. **(C)** White Blood Cell Count (WBC) Levels. **(D)** Neutrophil Percentage (Neu%) Levels.

8 trials using a random effects model reported the CRP levels between the intervention and control groups. The results demonstrated that the intervention group had a significantly lower CRP level than the control group [MD = −6.50, 95% CI (−9.97, −3.03), *P* = 0.0002]. However, it is essential to note the considerable heterogeneity (*I*^2^ = 93%, *P* < 0.00001) (Figure 5A).

##### Procalcitonin (PCT) levels

5 trials used a random effects model to compare the PCT levels between the intervention and control groups. The results demonstrated that the intervention group had a significantly lower PCT level than the control group [MD = −0.81, 95% CI (−1.21, −0.40), *P* < 0.0001]. However, substantial heterogeneity was observed (*I*^2^ = 95%, *P* < 0.00001) (Figure 5B).

##### White blood cell count (WBC) levels

4 trials reported the WBC levels between the intervention and control groups using a random effects model. The results demonstrated that the intervention group had a significantly lower WBC level than the control group [MD = −6.52, 95% CI (−8.31, −4.73), *P* < 0.00001], The included trials exhibited moderate heterogeneity (*I*^2^ = 64%, *P* = 0.04) (Figure 5C).

##### Neutrophil percentage (Neu%) levels

3 trials reported the Neu% levels between the intervention and control groups using a random effects model. The results demonstrated that the intervention group had a significantly lower WBC level than the control group [MD = −6.66, 95% CI (−8.96, −4.36), *P* < 0.00001], Moderate heterogeneity was observed among the trials (*I*^2^ = 73%, *P* = 0.02) (Figure 5D).

#### 3.4.3 Pulmonary symptoms

##### Cough duration

**FIGURE 6:**
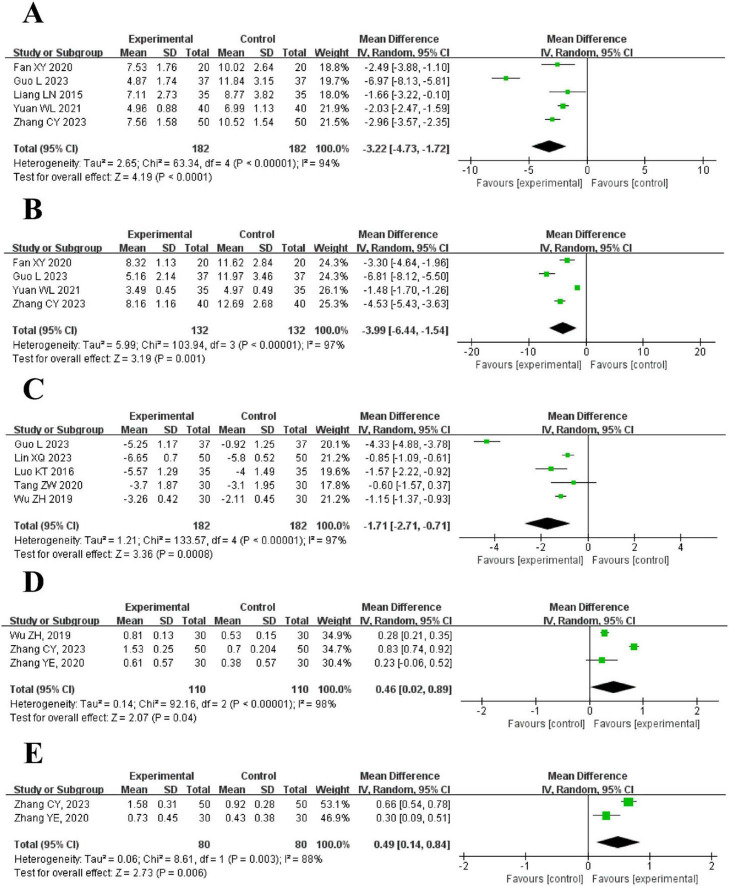
The forest plot of pulmonary symptoms compared to acupuncture plus CT vs. CT. **(A)** Cough Duration. **(B)** Duration Until Disappearance of Rales. **(C)** Clinical pulmonary infection score (CPIS). **(D)** Forced vital capacity (FVC). **(E)** Forced expiratory volume in 1 s (FEV_1_).

5 trials reported cough duration showed that the intervention group had a significantly shorter cough duration than the control group [MD = −3.22, 95% CI (−4.73, −1.72), *P* < 0.0001] with high heterogeneity (*I*^2^ = 94%, *P* < 0.00001). As shown in Figure 6A.

##### Duration until disappearance of rales

4 trials indicated that the duration until the disappearance of rales was shorter in the intervention group [MD = −3.99, 95% CI (−6.44, −1.54), *P* = 0.001]. High heterogeneity was found (*I*^2^ = 97%, *P* < 0.00001). As shown in Figure 6B.

##### Clinical pulmonary infection score (CPIS)

5 trials reported CPIS showed that the intervention group had a lower score than the control group [MD = −1.71, 95% CI (−2.71, −0.71), *P* = 0.0008], with high heterogeneity (*I*^2^ = 97%, *P* < 0.00001). As shown in Figure 6C.

##### Forced vital capacity (FVC)

3 trials showed that FVC was higher in the intervention group [MD = 0.46, 95% CI (0.02, 0.89), *P* = 0.04], with high heterogeneity (*I*^2^ = 98%, *P* < 0.00001). As shown in Figure 6D.

##### Forced expiratory volume in 1 s (FEV_1_)

2 trials found that FEV_1_ was higher in the intervention group [MD = 0.49, 95% CI (0.14, 0.84), *P* = 0.006], with high heterogeneity (*I*^2^ = 88%, *P* = 0.003). As shown in Figure 6E.

#### 3.4.4 NIHSS

**FIGURE 7:**
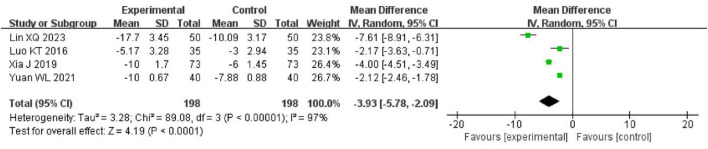
The forest of NIHSS compared to acupuncture plus CT vs. CT.

4 trials reported the NIHSS between the intervention and control groups using a random-effects model. The results demonstrated that the intervention group had a significantly lower NIHSS than the control group [MD = −3.93, 95% CI (−5.78, −2.09), *P* < 0.00001], with high heterogeneity (*I*^2^ = 97%, *P* < 0.00001). As shown in Figure 7.

#### 3.4.5 Duration of antibiotic use

**FIGURE 8:**
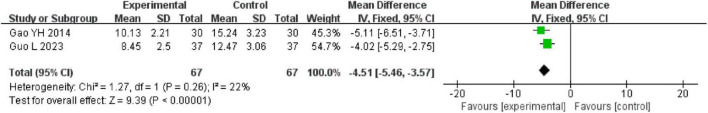
The forest plot of effective rate compared to acupuncture plus CT vs. CT.

2 trials compared the duration of antibiotic use between the intervention group and the control group. The results of the meta-analysis showed that the intervention group had a significantly shorter duration of antibiotic use than the control group [MD = −4.51, 95% CI (−5.46, −3.57), *P* < 0.00001]. The studies demonstrated low heterogeneity and no statistically significant difference (*I*^2^ = 22%, *P* = 0.26). As shown in Figure 8.

#### 3.4.6 Publication bias

**FIGURE 9:**
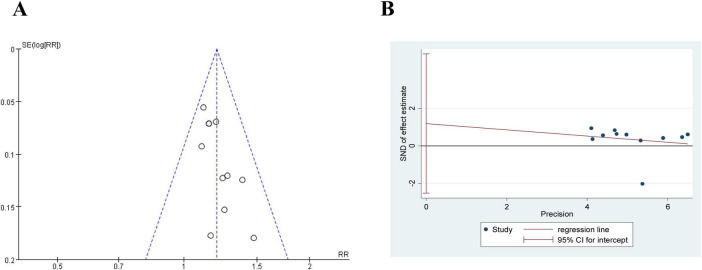
The publication bias of effective rate in comparing acupuncture plus CT vs. CT. **(A)** Funnel plots. **(B)** Egger’s tests.

As shown in Figure 9A, the funnel plot shows that the research results are biased toward one side of the funnel plot, which may suggest publication bias. We used Egger’s tests to evaluate publication bias in the included studies. As shown in Figure 9B, Egger’s tests indicated that there was no evidence of publication bias regarding the effective rate (*P* = 0.489).

#### 3.4.7 Safety

Although no adverse effects associated with acupuncture treatment were reported in the selected literature, potential adverse effects cannot be excluded entirely.

## 4 Discussion

### 4.1 Summary of the results

This meta-analysis included 16 trials involving 1,125 patients, comparing the effects of acupuncture therapy to CT. Our findings indicate that patients who underwent acupuncture treatment had a higher efficacy rate, lower levels of inflammation (WBC, CRP, Neu%, and PCT), and a reduced duration of antibiotic use. Additionally, acupuncture improved pulmonary conditions such as FVC and FEV_1_, shortened the duration of coughing, quicker the disappearance of rales, and resulted in a lower CPIS and NIHSS than those who did not receive acupuncture treatment. Additionally, although no adverse events related to acupuncture were reported, the possibility of adverse reactions cannot be excluded entirely due to the lack of definitive reporting in the included trials. Finally, due to the low-quality evidence, more rigorous studies are needed in the coming years to confirm these findings.

### 4.2 The effect of acupuncture on SAP

Our study findings indicate that the majority of patients with SAP included in the study were elderly individuals aged 60 years and older, a demographic characterized by advanced age, which is a major risk factor for SAP (40). Advanced-age stroke patients often present with multiple comorbidities and require prolonged bed rest, posing various challenges in clinical management. The misuse of antibiotics has led to the emergence of antimicrobial resistance, while post-stroke immunosuppression further complicates the clinical treatment of SAP (41, 42). Moreover, there is a lack of consensus regarding the definition and diagnostic criteria for SAP, resulting in inadequacies and delays in its prevention, diagnosis, and treatment in clinical practice (8). This may contribute to decreased treatment efficacy and poor prognosis for patients. In contrast to the limitations of antibiotic therapy, acupuncture offers the advantage of minimal side effects (43). In our study, the effective rate in the treatment group was significantly higher than that in the control group, with a statistically meaningful difference (RR = 1.20, 95% CI [1.13, 1.27], *P* < 0.00001). Furthermore, there was no significant heterogeneity between the two groups (*P* = 0.79, *I*^2^ = 0%), indicating that acupuncture interventions positively impact the clinical treatment efficacy of SAP.

Recent studies have indicated a close relationship between SAP and systemic immune dysregulation induced by post-stroke brain injury. Stroke can trigger the release of various immune mediators, including IL-1β, TNF-α, calcitonin gene-related peptides, neuropeptides, and vasoactive intestinal peptides. These immune mediators activate specific signaling pathways that lead to systemic immune suppression, exacerbating neurological deficits post-stroke, diminishing host immune function, and increasing susceptibility to infections as well as issues related to antibiotic resistance (44, 45). CRP and PCT have been identified as independent risk factors influencing pulmonary infections in stroke patients, and elevated levels of CRP are considered a significant risk factor for mortality in elderly patients with SAP (46, 47). During infection or trauma, CRP levels can rise rapidly, demonstrating high sensitivity in the early stages of infection (48). Furthermore, increased serum PCT levels correlate positively with the severity of neuroinflammation and injury, as well as with adverse outcomes in patients with intracerebral hemorrhage (49). WBC and Neu% are commonly used clinical inflammatory markers that participate in the progression of inflammatory lesions. In patients with pneumonia, these markers are typically elevated, aiding in the assessment of the condition and infection severity in SAP patients (50). Results from included studies demonstrate that, compared to conventional Western Medical treatment alone, acupuncture combined with conventional therapy significantly reduces the levels of CRP, WBC, Neu%, and PCT, suggesting that acupuncture may improve patient conditions by lowering inflammatory factor levels. This phenomenon may be attributed to acupuncture’s ability to regulate systemic Qi and blood circulation by stimulating specific acupoints, harmonizing organ function, thereby alleviating inflammatory responses and reducing pneumonia symptoms (51, 52). Acupuncture stimulation can affect nerve endings, activate the nervous system, and modulate immune function through neuro-endocrine-immune network mechanisms, inhibiting the release of inflammatory factors. Additionally, acupuncture can stimulate the body to release endogenous anti-inflammatory substances, such as β-endorphins, thereby suppressing the inflammatory process and alleviating pulmonary inflammation (53).

Neurological damage resulting from stroke can significantly impair respiratory muscle function, consequently affecting lung capacity and airway clearance (54). FVC and FEV_1_ are critical indicators for assessing pulmonary function. Stroke patients often exhibit marked declines in these parameters, leading to inadequate pulmonary ventilation and an increased risk of infection (55). Furthermore, the cough reflex in stroke patients may be suppressed, resulting in a shortened cough duration that hampers the ability to clear airway secretions, thereby exacerbating the risk of pulmonary infections. CPIS integrates clinical, microbiological, and radiological indicators, effectively reflecting the severity of pulmonary infections; higher scores indicate more severe infections (56). The duration of the disappearance of rales is also a crucial indicator for assessing the progression of pulmonary infections, with prolonged duration potentially signaling worsening conditions. Therefore, timely assessment of pulmonary function and infection risk in stroke patients, along with proactive preventive and therapeutic measures, is essential for reducing the incidence of SAP and improving patient outcomes. Our study demonstrates that the acupuncture combined treatment group shows superior efficacy in alleviating pulmonary symptoms compared to the control group, suggesting that acupuncture may be beneficial in managing SAP by improving symptoms and signs of pulmonary infection.

The NIHSS score greater than 15 is recognized as an independent risk factor for the development of SAP (57). Higher NIHSS scores indicate more severe neurological impairment, which correlates with an increased risk of pulmonary infections (58). Previous studies have demonstrated that acupuncture can enhance immune function and promote neurological recovery (59, 60). Our meta-analysis reveals that the acupuncture treatment group significantly reduces NIHSS scores, indicating improved neurological outcomes. Furthermore, research indicates prolonged antibiotic therapy may disrupt the gut microbiota, thereby increasing the risk of infections (61, 62). Our findings suggest that acupuncture as an adjunct therapy can significantly reduce the duration of antibiotic use by an average of 4.51 days, which may help mitigate antibiotic-related side effects and the risk of antibiotic resistance.

### 4.3 Mechanism of acupuncture on SAP

After a stroke, inflammatory responses aid tissue healing and removing necrotic cells. However, when inflammation becomes excessive, it can cause additional harm (4). Research indicates that the BL13 acupoint modulates pneumonia by targeting multiple genes, such as FCER2, IL4R, FASLG, and others, to modulate cytokine signaling in the immune system (63). Additionally, it has been shown that electroacupuncture at BL13 down-regulates the lung index and serum TNF-α levels and up-regulates serum IL-10 levels in mice with viral pneumonia (64). Additionally, electroacupuncture at GV20 and ST36 demonstrates anti-inflammatory effects by inhibiting the release of cytokines, such as localized TNFα, and suppressing the expression of heat shock protein 70 (HSP70) (65). Moreover, electroacupuncture at GB20 activates the cerebral cortex regions associated with swallowing, enhancing functional connectivity and brain remodeling and improving swallowing function (66). Besides, acupuncture at GV20, ST36, and LI4 has been shown to benefit the rehabilitation of the central nervous system in patients recovering from ischemic stroke (18). Finally, electroacupuncture at CV23 improves swallowing function in mouse models of post-stroke dysphagia by facilitating motor cortex inputs to the nucleus tractus solitaries through the parabrachial nuclei (67).

In summary, acupuncture on specific acupoints, such as BL13, GV20, ST36, LI11, GB20, and CV23, exerts anti-inflammatory effects through multiple mechanisms, which supports the effectiveness of acupuncture for SAP. These findings provide a solid theoretical basis for further clinical applications and research.

### 4.4 Implications for future research and practice

As secondary to stroke, the effective management of the primary condition (stroke) is crucial in the treatment of SAP. Standard medical management should be regarded as the primary therapeutic option in both experimental and control groups, with detailed implementation protocols comprehensively documented within the study.

The specifics of acupuncture treatment protocols vary across studies; however, many of the included trials inadequately reported essential information, failing to meet all Standards for Reporting Interventions in Clinical Trials of Acupuncture (STRICTA) criteria (68). This lack of detail limits the reader’s ability to assess the quality of acupuncture interventions. Syndrome differentiation, a common practice in clinical acupuncture, poses challenges in replication and evaluation due to its individualized diagnostic approach. Therefore, standardized acupuncture treatment protocols should remain the preferred choice in clinical trials. Nevertheless, numerous studies have demonstrated the efficacy of syndrome-based treatments, highlighting the importance of balancing standardized interventions with flexible, personalized approaches.

In summary, future research should focus on increasing sample sizes and employing more rigorous and effective experimental designs to evaluate the efficacy and safety of acupuncture for SAP. Sample size calculations should be based on standardized formulas and previous research findings. To enhance methodological quality, future trials should: (1) utilize more standardized approaches for syndrome differentiation and acupoint selection; (2) classify recruited patients more meticulously according to the severity and duration of SAP; (3) adhere to STRICTA guidelines and Consolidated Standards of Reporting Trials (CONSORT) statements (69); (4) implement appropriate blinding methods; and (5) prioritize follow-up assessments as a critical component of future studies.

### 4.5 Limitations of study

This systematic review and meta-analysis is subject to several limitations. Firstly, the included studies exhibited considerable variability in quality. Secondly, there was a lack of standardized experimental designs and interventions across the studies, with notable discrepancies in acupoint selection, needle retention time, treatment frequency, and session duration. Thirdly, the incomplete reporting of essential aspects of the experimental design, such as the specific acupuncture techniques employed and the researchers’ backgrounds, significantly hampers our ability to assess the overall quality of the literature. Lastly, potential publication bias necessitates caution in the interpretation of the results of this study.

## 5 Conclusion

The research found that acupuncture has a positive effect in treating SAP. However, owing to the low-quality evidence, additional studies are warranted to corroborate these findings in the coming years.

## Data Availability

The datasets presented in this study can be found in online repositories. The names of the repository/repositories and accession number(s) can be found in this article/Supplementary material.
