# Supplementary material for: Efficacy of acupuncture for stroke-associated pneumonia: a systematic review and meta-analysis
Source: Front Med (Lausanne). 2025 Mar 3;12:1440121. doi: 10.3389/fmed.2025.1440121 (PMC11911211; doi:10.3389/fmed.2025.1440121)
Supplement: Supplementary file 1 [file Supplementary_file_1.pdf]

Efficacy of acupuncture for Stroke-Associated Pneumonia: A systematic review and meta-analysis

Supplementary Material: Search strategy

**Database**		**Search strategy**
	#1	"acupuncture"[MeSH Terms]
	#2	("acupuncture"[Title/Abstract] OR "Acupuncture Therapy"[Title/Abstract] OR "Auricular points"[Title/Abstract] OR "Ear Acupuncture"[Title/Abstract] OR "electroacupuncture"[Title/Abstract] OR "electro-acupuncture"[Title/Abstract] OR "auricular"[Title/Abstract] OR "acusector"[Title/Abstract] OR "needle"[Title/Abstract] OR "fire needle"[Title/Abstract] OR "Acupoint catgut embedding"[Title/Abstract] OR "Bleeding"[Title/Abstract] OR "Acupoint injection"[Title/Abstract] OR "Needle knife"[Title/Abstract] OR "Plum-blossom needle"[Title/Abstract])
	#3	#1 OR #2
	#4	" Stroke"[MeSH Terms]
Pubmed	#5	"Strokes"[Title/Abstract] OR "Cerebrovascular Accident"[Title/Abstract] OR "Cerebrovascular Accidents"[Title/Abstract] OR "CVA"[Title/Abstract] OR "CVAs"[Title/Abstract] OR "Cerebrovascular Apoplexy"[Title/Abstract] OR "Brain Vascular Accident"[Title/Abstract] OR "Brain Vascular Accidents"[Title/Abstract] OR "Cerebrovascular Stroke"[Title/Abstract] OR "Cerebrovascular Strokes"[Title/Abstract] OR "Apoplexy"[Title/Abstract] OR "Cerebral Stroke"[Title/Abstract] OR "Cerebral Strokes"[Title/Abstract]
	#6	#4 OR #5
	#7	" Pneumonia"[MeSH Terms]
	#8	"Pneumonias"[Title/Abstract] OR "Lobar Pneumonia"[Title/Abstract] OR "Lobar Pneumonias"[Title/Abstract] OR "Experimental Lung Inflammation"[Title/Abstract] OR "Experimental Lung Inflammations"[Title/Abstract] OR "Lung Inflammation,

		Experimental"[Title/Abstract] OR "Pneumonitis"[Title/Abstract] OR "Pneumonitides"[Title/Abstract] OR "Pulmonary Inflammation"[Title/Abstract] OR "Pulmonary Inflammations"[Title/Abstract] OR "Lung Inflammation"[Title/Abstract] OR "Lung Inflammations"[Title/Abstract]
	#9	#7 OR #8
	#10	("randomized controlled trial"[Publication Type] OR "controlled clinical trial"[Publication Type] OR "randomized"[Title/Abstract] OR "placebo"[Title/Abstract] OR "clinical trials as topic"[MeSH Terms] OR "randomly"[Title/Abstract] OR "trial"[Title]) NOT ("animals"[MeSH Terms] NOT "humans"[MeSH Terms])
	#11	#3 AND #6 AND #9 AND #10
Web of Science	#1	TS = ("Strokes" OR "Cerebrovascular Accident" OR "Cerebrovascular Accidents" OR "CVA" OR "CVAs" OR "Cerebrovascular Apoplexy" OR "Brain Vascular Accident" OR "Brain Vascular Accidents" OR "Cerebrovascular Stroke" OR "Cerebrovascular Strokes" OR "Apoplexy" OR "Cerebral Stroke" OR "Cerebral Strokes")
	#2	TS = ("Pneumonias" OR "Lobar Pneumonia" OR "Lobar Pneumonias" OR "Experimental Lung Inflammation" OR "Experimental Lung Inflammations" OR "Lung Inflammation, Experimental" OR "Pneumonitis" OR "Pneumonitides" OR "Pulmonary Inflammation" OR "Pulmonary Inflammations" OR "Lung Inflammation" OR "Lung Inflammations")
	#3	TS = ("Acupuncture" OR "Acupuncture Therapy" OR "Auricular points" OR "Ear Acupuncture" OR Electroacupuncture OR "Electro-acupuncture" OR "Auricular OR "Acusector" OR "Needl" OR "Fire needle" OR "Acupoint catgut embedding" OR Bleeding OR "Acupoint injection" OR "Needle knife" OR "Plum-blossom needle")
	#4	:TS = ("Randomized" OR Trials OR Placebo OR "Controlled Clinical Trial" OR "Randomized Controlled Trial" OR "Clinical Trials as Topic" OR "Randomly" OR "Trial")
	#5	#1 AND #2 AND #3 AND #4
Cochrane Library	#1	(Stroke):ti,ab,kw OR (Strokes):ti,ab,kw OR (Cerebrovascular Accident):ti,ab,kw OR (Cerebrovascular Accidents):ti,ab,kw OR (CVA):ti,ab,kw OR (CVAs):ti,ab,kw OR (Cerebrovascular Apoplexy):ti,ab,kw OR (Apoplexy, Cerebrovascular):ti,ab,kw OR (Brain

		Vascular Accident):ti,ab,kw OR (Brain Vascular Accidents):ti,ab,kw OR (Cerebrovascular Stroke):ti,ab,kw OR (Cerebrovascular Strokes):ti,ab,kw OR (Apoplexy):ti,ab,kw OR (Cerebral Stroke):ti,ab,kw OR (Cerebral Strokes):ti,ab,kw OR (Acute Stroke):ti,ab,kw OR (Acute Strokes):ti,ab,kw OR (Acute Cerebrovascular Accident):ti,ab,kw OR (Acute Cerebrovascular Accidents):ti,ab,kw
	#2	(Pneumonia):ti,ab,kw OR (Pneumonias):ti,ab,kw OR (Lobar Pneumonia):ti,ab,kw OR (Lobar Pneumonias):ti,ab,kw OR (Experimental Lung Inflammation):ti,ab,kw OR (Experimental Lung Inflammations):ti,ab,kw OR (Pneumonitis):ti,ab,kw OR (Pneumonitides):ti,ab,kw OR (Pulmonary Inflammation):ti,ab,kw OR (Pulmonary Inflammations):ti,ab,kw OR (Lung Inflammation):ti,ab,kw OR (Lung Inflammations):ti,ab,kw
	#3	(Acupuncture):ti,ab,kw OR (Acupuncture Therapy):ti,ab,kw OR (Auricular points):ti,ab,kw OR (Ear Acupuncture):ti,ab,kw OR (Electroacupuncture):ti,ab,kw OR (Electro-acupuncture):ti,ab,kw OR (Auricular):ti,ab,kw OR (Acusector):ti,ab,kw OR (Needle):ti,ab,kw OR (Fire needle):ti,ab,kw OR (Acupoint catgut embedding):ti,ab,kw OR (Bleeding):ti,ab,kw OR (Acupoint injection):ti,ab,kw OR (Needle knife):ti,ab,kw OR (Plum-blossom needle):ti,ab,kw
	#4	(randomized):ti,ab,kw OR (randomly):ti,ab,kw OR (placebo):ti,ab,kw OR (tiral):ti,ab,kw
	#5	#1 AND #2 AND #3 AND #4
Embase	#1	('Acupuncture':ab,ti OR 'Acupuncture Therapy':ab,ti OR 'Auricular points':ab,ti OR 'Ear Acupuncture':ab,ti OR 'Electroacupuncture':ab,ti OR 'Electro-acupuncture':ab,ti OR 'Auricular':ab,ti OR 'Acusector':ab,ti OR 'Needl':ab,ti OR 'Fire needle':ab,ti OR 'Acupoint catgut embedding':ab,ti OR 'Bleeding':ab,ti OR 'Acupoint injection':ab,ti OR 'Needle knife':ab,ti OR 'Plum-blossom needle':ab,ti)
	#2	('Strokes':ab,ti OR 'Cerebrovascular Accident':ab,ti OR 'Cerebrovascular Accidents':ab,ti OR 'CVA':ab,ti OR 'CVAs':ab,ti OR 'Cerebrovascular Apoplexy':ab,ti OR 'Apoplexy, Cerebrovascular':ab,ti OR 'Brain Vascular Accident':ab,ti OR 'Brain Vascular Accidents':ab,ti OR 'Cerebrovascular Stroke':ab,ti OR 'Cerebrovascular Strokes':ab,ti OR 'Apoplexy':ab,ti OR 'Cerebral Stroke':ab,ti OR 'Cerebral Strokes':ab,ti OR 'Acute Stroke':ab,ti OR 'Acute Strokes':ab,ti OR 'Acute Cerebrovascular

		Accident':ab,ti OR 'Acute Cerebrovascular Accidents':ab,ti)
	#3	('Pneumonia':ab,ti OR 'Pneumonias':ab,ti OR 'Lobar Pneumonia':ab,ti OR 'Lobar Pneumonias':ab,ti OR 'Experimental Lung Inflammation':ab,ti OR 'Experimental Lung Inflammations':ab,ti OR 'Pneumonitis':ab,ti OR 'Pneumonitides':ab,ti OR 'Pulmonary Inflammation':ab,ti OR 'Pulmonary Inflammations':ab,ti OR 'Lung Inflammation':ab,ti OR 'Lung Inflammations':ab,ti)
	#4	('randomized controlled trial':ab,ti OR ('controlled clinical trial' OR 'randomized':ab,ti OR 'randomly':ab,ti OR 'trial':ab,ti OR 'placebo':ab,ti OR 'clinical article':ab,ti OR 'clinical trial':ab,ti OR 'controlled study':ab,ti OR 'major clinical study':ab,ti OR 'double blind procedure':ab,ti OR 'multicenter study':ab,ti OR 'single blind procedure':ab,ti OR 'crossover procedure':ab,ti))
	#5	#1 AND #2 AND #3 AND #4
China National Knowledge Infrastructure （CNKI）	#1	(SU = '针灸' OR SU = '针刺' OR SU = '电针' OR SU = '温针灸' OR SU = '火针' OR SU = '头针' OR SU = '皮肤针' OR SU = '刺血 ' OR SU = '毫针' OR SU = '针刀')
	#2	(SU = '卒中相关性肺炎' OR SU = '中风相关性肺炎' OR SU = '卒中后肺炎' OR SU = '中风后肺炎' OR SU = '脑卒中并发肺炎' OR SU = '中风并发肺炎' OR SU = '感染性肺炎' OR SU = '肺部感染' OR SU = '肺炎')
	#3	(SU = '卒中’ OR SU = '中风' OR SU = '脑卒中' OR SU = '脑中风' OR SU = '脑血管意外' OR SU = '脑梗死' OR SU = '脑出血')
	#4	(SU='随机' OR FT='随机' OR FT='试验' OR FT='观察' OR FT='疗效评价')
	#5	#1 AND #2 AND #3 AND #4
Database for Chinese Technical Periodicals (VIP)	#1	M=（针灸 OR 针刺 OR 电针 OR 温针灸 OR 火针 OR 头针 OR 皮肤针 OR 刺血 OR 毫针 OR 针刀）
	#2	M=（卒中相关性肺炎 OR 中风相关性肺炎 OR 卒中后肺炎 OR 中风后肺炎 OR 脑卒中并发肺炎 OR 中风并发肺炎 OR 感染性肺炎 OR 肺部感染 OR 肺炎）
	#3	M=（卒中 OR 中风 OR 脑卒中 OR 脑中风 OR 脑血管意外 OR 脑梗死 OR 脑出血）
	#4	M=（试验 OR 观察 OR 随机 OR 疗效评价）

	#5	#1 AND #2 AND #3 AND #4
Wanfang Database	#1	（针灸 OR 针刺 OR 电针 OR 温针灸 OR 火针 OR 头针 OR 皮肤针 OR 刺血 OR 毫针 OR 针刀）
	#2	（卒中相关性肺炎 OR 中风相关性肺炎 OR 卒中后肺炎 OR 中风后肺炎 OR 脑卒中并发肺炎 OR 中风并发肺炎 OR 感染性肺炎 OR 肺部感染 OR 肺炎）
	#3	（卒中 OR 中风 OR 脑卒中 OR 脑中风 OR 脑血管意外 OR 脑梗死 OR 脑出血）
	#4	（试验 OR 观察 OR 随机 OR 疗效评价）
	#5	#1 AND #2 AND #3 AND #4
Chinese Biomedical Literature Database	#1	（针灸 OR 针刺 OR 电针 OR 温针灸 OR 火针 OR 头针 OR 皮肤针 OR 刺血 OR 毫针 OR 针刀）
	#2	（卒中相关性肺炎 OR 中风相关性肺炎 OR 卒中后肺炎 OR 中风后肺炎 OR 脑卒中并发肺炎 OR 中风并发肺炎 OR 感染性肺炎 OR 肺部感染 OR 肺炎）
	#3	（卒中 OR 中风 OR 脑卒中 OR 脑中风 OR 脑血管意外 OR 脑梗死 OR 脑出血）
	#4	（试验 OR 观察 OR 随机 OR 疗效评价）
	#5	#1 AND #2 AND #3 AND #4
