# Supplementary material for: Efficacy of acupuncture for stroke-associated pneumonia: a systematic review and meta-analysis
Source: Front Med (Lausanne). 2025 Mar 3;12:1440121. doi: 10.3389/fmed.2025.1440121 (PMC11911211; doi:10.3389/fmed.2025.1440121)
Supplement: Supplementary file 2 [file Supplementary_file_2.docx]

| Author, year | Control group treatment |
| --- | --- |
| Zhang CY, 2023 | Symptomatic treatment（Improve cerebral circulation, reduce blood pressure, relieve cough, resolve phlegm, fight against infections, administer low-flow oxygen inhalation, and perform nebulization.  ） + Pulmonary Rehabilitation Treatment (Breathing Control Training, twice a day, 30 minutes each time; Respiratory Muscle Training, Twice a day, 15 minutes each time). |
| Lin XQ, 2023 | Cefoperazone Sodium Sulbactam Sodium + Pulmonary Rehabilitation (lip-contraction breathing exercise, 3-4times a day, 10 minutes each time + diaphragmatic breathing exercise, 5-15 minutes each time, 2-3 times a day). |
| Guo YF,2023 | Symptomatic treatment (Active control of blood pressure and blood sugar and other primary diseases, oxygen inhalation, sputum aspiration, reducing intracranial pressure, anti-platelet aggregation, anti-coagulation, maintenance of water and electrolyte balance, nutritional support, control of infection)+ positional management, cough instruction, respiratory training, 10 minutes at a time, three times a day. |
| Guo L,2023 | Aspirin enteric-coated tablets (100mg, Po, QD), calcium Atorvastatin tablets (20mg, Po, QD), and calcium tablets (20mg, Po, QD) were used to reduce blood lipids and stabilize arteriosclerosis plaque Edaravone-right camphor for injection 15 ml of concentrated solution was given intravenously for 12 hours Sodium butylphthalide chloride injection (100mL IV drip, 12h each time) was given according to the sputum culture drug sensitivity test for the aspiration pneumonia. |
| Ma YQ,2022 | Symptomatic treatment + comprehensive rehabilitation measures (respiratory training, exercise training, instruction in expectoration, and Ultrashort wave therapy). |
| Yuan WL, 2021 | Anti-infective (cefoperazone sodium sulbactam sodium intravenous drip, 3 days does not work, then adjusted according to sputum culture and drug sensitivity results), antipyretic (temperature 38.5 ℃ or more intramuscular injection with compound aminobarbital injection), nasal catheter oxygen (nebulization or antispasmodic asthma, if necessary), sputum oral aminobromide hydrochloride tablets of the treatment of patients with pronounced stridor. |
| Tang ZW, 2020 | Anti-infective therapy: cefoperazone sodium and sulbactam sodium for injection (1.5g), intravenous drip, 3 g per day, with 5% glucose injection or 0.55% glucose. 9% sodium chloride injection was diluted by intravenous drip every 12 hours, and those who had no effect after 3 days were treated with anti-infection therapy according to sputum culture and drug sensitivity test for 10 days. The patients above 38.5℃ were given intramuscular injections of compound amobarbital (2 ml), 2 ml each time (not more than 3 times a day), body temperature was closely observed, and the patients with obvious wheezing were treated with oxygen inhalation through nasal catheter, 4 patients with more sputum were treated with ambroxol hydrochloride tablets (30mg), 30mg, Po, Tid for 10 days. |
| Zhang YE, 2020 | Standardized treatment for stroke (dehydration and lowering of cranial pressure, anticoagulation, antiplatelet, symptomatic supportive therapy for pneumonia) + extracorporeal diaphragmatic pacing + reasonable use of antibiotics to fight infection, expectoration, asthma, non-invasive assisted ventilation, etc., close observation of blood pressure, blood glucose, correction of electrolyte disorders. |
| Fan XY, 2020 | Symptomatic treatment (antihypertensive, hypoglycemic, nutritive neurologic, improve cerebral circulation and other medications, at the same time give low-flow oxygen, regulate water electrolytes) + respiratory control (lip-contracting respiration, abdominal respiration) + respiratory muscle training (diaphragm resistance, inspiratory muscle training). |
| Xia J, 2019 | Routine Management: This includes dietary interventions, feeding training, oral care, turning, and back-patting to help expel sputum.  Dietary Guidelines: Start with easy foods, eating in a semi-recumbent position with the head elevated 15° to 30°, or sitting upright. Eat slowly and maintain this position for 30 minutes after meals.  Swallowing Rehabilitation: This involves muscle strength and sensory training.  Muscle Strength Training: Perform exercises for the tongue and oral muscles (e.g., tongue extension, scraping, rolling) 10 times twice a day.  Sensory Training: Use cotton balls with varying temperatures to alternately stimulate parts of the oral cavity and pharynx for 20 seconds each, repeating 10 times. |
| Wu ZH, 2019 | Basic therapy and breathing training, which includes breathing control (lip-contraction breathing and abdominal breathing) and respiratory muscle training (diaphragm resistance training and inspiratory muscle training). |
| Luo KT, 2016 | Symptomatic treatment (antiplatelet aggregation, vasodilation, improvement of cerebral metabolism, nutrition of brain cells, anti-infection, etc.) |
| Liu YC, 2016 | Swallowing training, physical cooling, sputum vibration, brain cell nutrition, blood pressure and blood sugar regulation, turnover and PAT back, and other symptomatic treatments. |
| Gu ZL, 2016 | Active treatment for primary and underlying diseases includes medications, posture adjustments, sputum suction, drainage, and nutritional support. Patients with hypoxemia may require low-flow oxygen inhalation or mechanical ventilation, with blood gas monitoring to ensure adequate oxygen levels.  Treatment usually starts with ceftazidime (1.2 g IV every 8 hours) or clindamycin (1.2-2.4 g IV twice daily). Aetiological and susceptibility tests are performed on pre-antibiotic specimens to guide treatment adjustments. |
| Liang LN, 2015 | Low-flow oxygen, 50ml of saline + 30mg of ambroxol hydrochloride, 2 times/d, intravenous drip, 50ml of saline + 0. 25g of aminophylline, 2 times/d, intravenous drip, and also appropriate antibiotic intravenous drip treatment selected according to the patient's sputum culture and drug sensitivity results. |
| Gao YH, 2014 | Improvement of cerebral circulation, nourishment of brain cells and symptomatic support, sputum culture, and drug sensitivity test results of antibiotic selection. |
